# Patterns and Trends of the Mortality From Bone Cancer in Pudong, Shanghai: A Population-Based Study

**DOI:** 10.3389/fonc.2022.873918

**Published:** 2022-05-16

**Authors:** Gui-Fen Ma, Qi-Yuan Bao, Hong-Yue Zhang, Yi-Chen Chen, Yue Zhang, Zhao-Yong Jiang, Xiao-Pan Li, Ju-Hua Zhang

**Affiliations:** ^1^ Department of Radiation Oncology, Zhongshan Hospital, Fudan University, Shanghai, China; ^2^ Center for Cancer Prevention and Treatment, Zhongshan Hospital, Fudan University, Shanghai, China; ^3^ Department of Orthopaedics, Ruijin Hospital Jiaotong University School of Medicine, Shanghai, China; ^4^ Shanghai Institute of Traumatology and Orthopedics, Shanghai Key Laboratory for Bone and Joint Diseases, Shanghai, China; ^5^ Department of Orthopedics, First Affiliated Hospital of Naval Medical University, Shanghai, China; ^6^ Office of Scientific Research and Information Management, Center for Disease Control and Prevention, Pudong New Area, Shanghai, China; ^7^ Office of Scientific Research and Information Management, Pudong Institute of Preventive Medicine, Pudong New Area, Shanghai, China; ^8^ Department of Epidemiology, Shanxi Medical University School of Public Health, Taiyuan, China; ^9^ Department of Spinal Surgery, Yongzhou Central Hospital, Hunan, China; ^10^ Department of Health Management Center, Zhongshan Hospital, Shanghai Medical College of Fudan University, Shanghai, China; ^11^ Department of Orthopedics in Traditional Chinese Medicine, Shanghai University of Medicine & Health Sciences, Affiliated Zhoupu Hospital, Shanghai, China; ^12^ Department of Social Medicine and Health Career Management, School of Public Administration, Fudan University, Shanghai, China; ^13^ Department of Social Medicine and Health Career Management, Shanghai Pudong Health Development Research Institute, Shanghai, China

**Keywords:** disease burden, bone cancer, trend analysis, aging, years of life lost, mortality, transitioning countries

## Abstract

**Introduction:**

The burden of cancer-related mortality of common malignancies has been reported worldwide. However, whether bone cancer (BC), as a highly aggressive and heterogeneous group of rare cancers, followed a similar or distinct epidemiological pattern during such process remains largely unknown. We aimed to analyze the mortality and the temporal trends of BC in relation to gender, age, and premature death in Shanghai, China.

**Methods:**

We conducted a population-based analysis of the mortality data of BC in Shanghai Pudong New Area (PNA) from 2005 to 2020. The epidemiological characteristics and long-term trends in crude mortality rates (CMRs), age-standardized mortality rates worldwide (ASMRWs), and rate of years of life lost (YLL) was analyzed using the Joinpoint regression program. The demographic and non-demographic factors affecting the mortality rate were evaluated by the decomposition method.

**Results:**

There are 519 BC-specific deaths accounting for 0.15% of all 336,823 deaths and 0.49% of cancer-specific death in PNA. The CMR and ASMRW of BC were 1.15/10^5^ person-year and 0.61/10^5^ person-year, respectively. The YLL due to premature death from BC was 6,539.39 years, with the age group of 60–69 years having the highest YLL of 1,440.79 years. The long-term trend of CMR, ASMRW, and YLL rate significantly decreased by −5.14%, −7.64%, and −7.27%, respectively, per year (all *p* < 0.05) in the past 16 years. However, the proportion of BC-specific death within the total cancer-specific death dropped to a plateau without further improvement since 2016, and a remarkable gender and age disparity was noticed in the observed reduction in mortality. Specifically, the elderly benefited less but accounted for a larger percentage of BC population in the last decades. Although the overall mortality of BC decreased, there was still a significant upward trend toward an increased mortality rate caused by the aging of the BC patients.

**Conclusion:**

Our study provides novel insights on the epidemiological characteristics and longitudinal dynamics of BC in a fast urbanization and transitioning city. As a rare disease affecting all ages, the burden of BC among the elderly emerged to form an understudied and unmet medical need in an aging society.

## Introduction

Bone cancer (BC) is a highly heterogeneous group of rare cancers, comprising over 50 different histologies (1). As a debilitating and metastasizing malignancy involving the musculoskeletal system, BC causes a significant disability and mortality and affects all age ranges compared to common cancer types. For example, as the three most common histologies, chondrosarcoma is usually diagnosed for people over 40 years of age, in contrast with Ewing sarcoma, which tends to impact children and teenagers (2). Osteosarcoma shows a bimodal distribution of the incidence, with a first peak occurring in the second decade and a second peak occurring in patients older than 60 years (3). Although the etiology and biology of bone cancer in the majority of the cases still remains unclear, the growth and development, germline genetics, somatic alterations, environmental exposure, and socioeconomic status (2) have all been proposed to be related to the predisposition and development of BC. Due to the fast transition of lifestyle behaviors, socioeconomic status, and healthcare system of modern society, the change in the spectrum of cancer mortality for several common cancer types has been widely reported. However, whether rare cancer types, such as BC, followed a similar or distinct epidemiological pattern during such process remains largely unknown. Therefore, a large-scale, population-based longitudinal study regarding the epidemiological change of BC is needed for better understanding and policy-making against such disease.

In the past several decades, China has experienced an outburst of economic growth, with Shanghai being a forerunner of such modernization process (4). Shanghai Pudong New Area (PNA) was established as and geared toward a national economic and technological development zone since the early 1990s. During the following decades, PNA became the largest and most populous region among the 16 districts, which represents one-fifth of the total population in Shanghai (4), with a geographic area of 1,210.41 km^2^ (467.34 mile^2^), and a registered permanent residency of more than 3.22 million (3). PNA is also the earliest area that began to establish modern healthcare infrastructure and to construct a sophisticated and reliable mortality registration system covering the total permanent resident population. PNA has witnessed the aging process in China’s economically developed areas, and with the migration of migrants, the population structure has undergone tremendous changes. Furthermore, PNA has established a death information registration system covering the whole population, which provides a reliable guarantee for analyzing death data (5, 6). Therefore, PNA might be an ideal representative to investigate the epidemiological profile and temporal changes in BC in the context of Shanghai as a fast modernization and transitioning society.

In this report, we aimed to comprehensively analyze the mortality data of BC collected from the Vital Statistics System of the entire population of PNA, Shanghai, China, from 2005 to 2020. We estimated the disease burden and mortality trend in the past decades to explore the epidemiological characteristics and the potential preventive strategies for BC in the future.

## Methods

### Data Source

According to the International Classification of Diseases 10th version (ICD-10), C40-41 refers to the primary malignant neoplasm that originated from bone and articular cartilage, also known as bone sarcoma. It accounts for 0.2% of all cancers and is one of the rare cancers. The most common subtypes of bone cancer are chondrosarcoma, osteosarcoma, chordoma, and Ewing sarcoma. In this report, data of BC (C40-C41)-related death of registered permanent residents from 2005 to 2020 were obtained from the Mortality Registration System of PNA, Shanghai. The complete population data were derived from the Public Security Bureau and the Statistics Bureau of PNA. Periodic evaluation and data cleaning are performed to maintain the integrity of the registration system according to standard guidelines. BC-specific deaths were classified according to the BC being an underlying cause of death according to the ICD-10. The causes of death were coded by clinicians according to the actual situation of patients and further checked by the local Centre for Disease Control and Prevention (CDC) (5). According to the 2000 Declaration of Helsinki, the study was performed and approved by the ethics committee of the Shanghai Pudong New Area Center for Disease Control and Prevention (IRB#2016-04-0586).

### Statistical Analyses

The crude mortality rates (CMRs) and age-standardized mortality rates by Segi’s world standard population (ASMRW) were shown as per 100,000 persons (/10^5^). The CMR and ASMRW were compared in gender by the Poisson approximation method and the Mantel–Haenszel test, respectively. Year of life lost (YLL) was calculated according to the original method described by Murray and Lopez. The formula of YLL adopted by the World Health Organization (WHO) (6–9).

Ages were calculated in the groups of 0–14 years, 15–29 years, 30–44 years, 45–59 years, 60–69 years, 70–79 years, and ≥80 years. The temporal trends of CMR, ASMRW, and YLL rate were calculated using the Joinpoint Regression Program 4.3.1.0 (National Cancer Institute, Bethesda, MD, USA) and expressed as average annual percent change (AAPC) with a corresponding 95% confidence interval (95% CI). The Z-test was used to assess whether the AAPC was statistically different from zero. The terms “increase” or “decrease” were used to describe a statistically significant (*p <*0.05) AAPC, while “stable” was used for non-significant trends (10).

The mortality rates of each year from 2006 to 2020, compared with the 2005 data, caused by demographic and non-demographic factors were estimated by the decomposition method, in which mortality rates were calculated and compared for each 5-year age group, from 0 to 4 to ≥85 years (8). All statistical analyses were conducted using SPSS (version 21.0; SPSS, Inc., Chicago, IL, USA) and R (version 3.4.3). Statistical significance was set at *p <*0.05.

## Results

### Baseline

**Figure 1:**
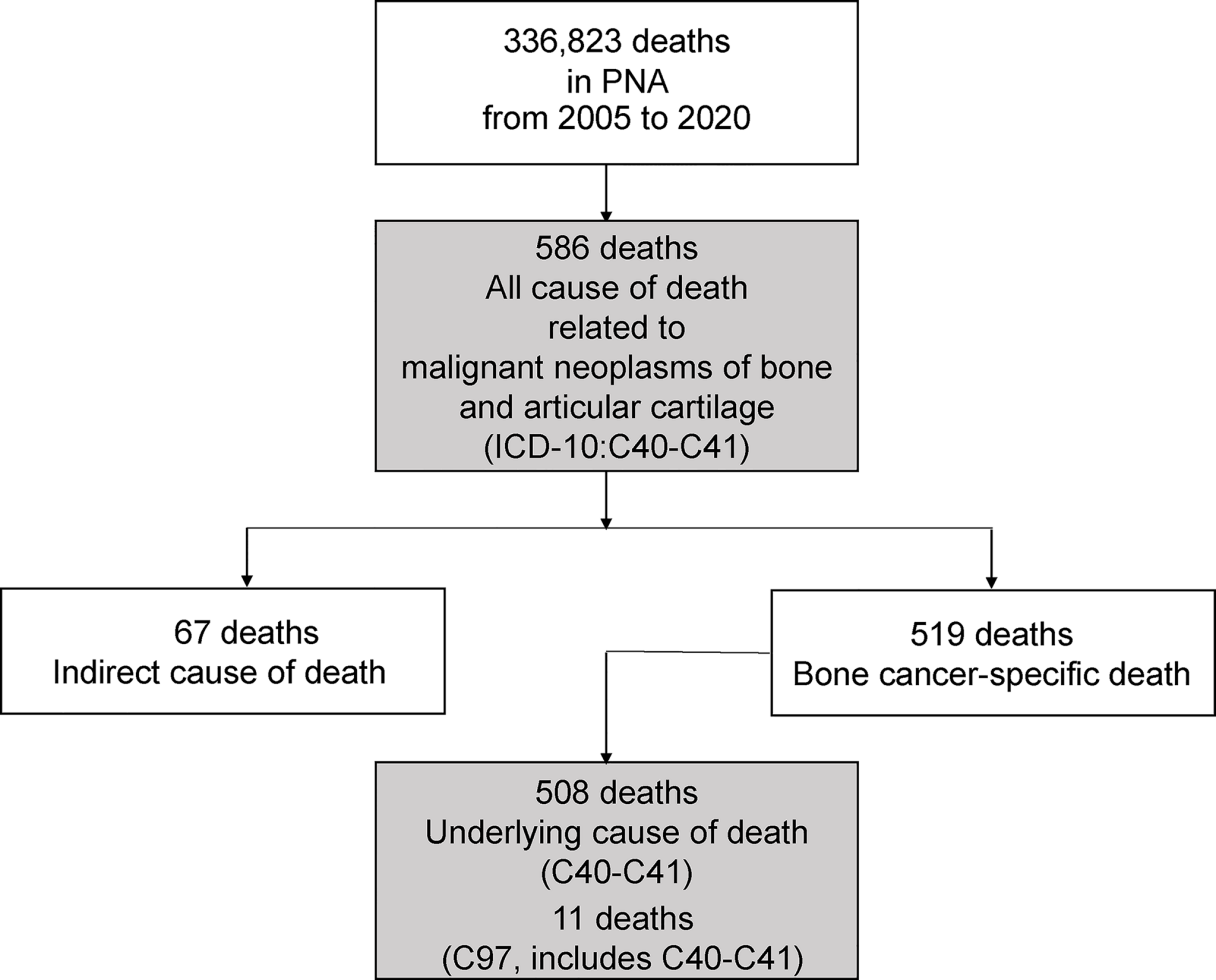
The flow chart of deaths from bone cancer in Shanghai Pudong New Area being include in this study.

A total of 519 BC-specific deaths from 586 BC-related deaths (**Supplementary Tables S1**–**S3**) were identified, accounting for 0.15% of all 336,823 deaths from 2005 to 2020 in Shanghai PNA, which included 508 underlying cause of death (C40–C41) and 11 death (C97, C40–C41) (**Figure 1**). There were 281 male (54.14%) and 238 female (45.86%) patients who died of BC. The median age and average age at death from BC were 71.82 years and 67.26 ± 18.62 years. The CMR and ASMRW of BC were 1.15/10^5^ person-year and 0.61/10^5^ person-year, respectively. The CMR and ASMRW were 1.25/10^5^ and 1.05/10^5^ person-years and 0.71/10^5^ and 0.52/10^5^ person-years in male and female patients, respectively. The CMR and ASMRW in male patients were higher than those in female patients (all *p* < 0.05) (**Table 1**).

**Table 1:** Baseline characteristics of deaths and burden in different genders and types of bone cancer during 2005–2020.

Characteristic	Deaths (n,%)	Age at years (mean ± SD)	Age at years (Median)	Age at years (Range)	CMR (/10^5^)	ASMRW (/10^5^)	YLL (years)	YLL rate (/10^5^)
Gender								
Male	281 (54.14)	65.46 ± 18.39	68.47	5.73-93.81	1.25	0.71	3604.24	16.01
Female	238 (45.86)	69.39 ± 18.71	75.16	12.62-94.19	1.05	0.52	2935.15	12.99
Periods								
2005–2008	169 (32.56)	61.11 ± 17.98	70.96	5.73-93.77	1.62	0.99	2216.12	21.27
2009–2012	137 (26.40)	66.70 ± 19.19	73.92	13.87-92.49	1.24	0.68	1757.79	15.96
2013–2016	106 (20.42)	66.57 ± 19.50	69.94	13.74-94.19	0.92	0.52	1346.94	11.69
2017–2020	107 (20.62)	70.49 ± 17.90	73.31	13.74-93.42	0.88	0.38	1218.55	10.03
Metastatic cancer								
All metastatic bone cancer	129 (24.86)	62.54 ± 20.78	67.35	12.62-94.19	0.29	0.19	1855.60	4.11
Metastatic bone cancer to the lung (C78.0)	53 (10.21)	57.38 ± 22.12	62.87	13.45-93.32	0.12	0.09	856.61	1.90
Metastatic bone cancer to the liver (C78.7)	11 (2.12)	64.36 ± 16.46	69.66	32.31-85.92	0.02	0.01	154.97	0.34
Metastatic bone cancer to the unknown sites								
47 (9.06)		66.13 ± 19.06	68.63	13.12-93.59	0.10	0.06	622.60	1.38
(C79) The main comorbidity in all causes of death								
Diseases of the respiratory system (J00–J99)	77 (14.84)	69.92 ± 17.92	72.61	13.45-93.54	0.17	0.08	880.54	1.95
Diseases of the circulatory system (I00–I99)	25 (4.82)	62.79 ± 23.42	73.66	13.12-90.99	0.06	0.04	350.40	0.78
Total bone cancer-specific death	519 (100.00)	67.26 ± 18.62	71.82	5.73-94.19	1.15	0.61	6539.39	14.50
Total all cause of death of the population	336,823 (/)	76.99 ± 14.44	80.45	0.00-116.39	746.71	279.39	3040514.99	6740.55

ASMRW, age-standardized mortality rate by Segi’s world standard population; CMR, crude mortality rate; YLL, years of life lost.

### Main Comorbidities of BC

**Figure 2:**
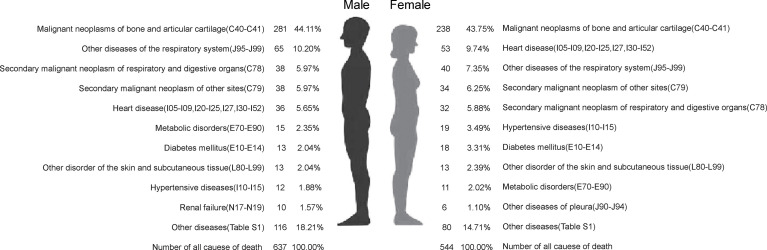
The top 10 of all causes of death of people who died from bone cancer in genders in Pudong New Area, Shanghai, China, 2005–2020.

The top 3 comorbidities in 281 male patients with BC as underlying cause of death were other diseases of the respiratory system (J95–J99) (10.20%), secondary malignant neoplasm of respiratory and digestive organs (C78) (5.97%), and secondary malignant neoplasm of other sites (C79) (5.97%). The top 3 comorbidities of 238 female patients whose underlying cause of death was BC were heart disease (I05–I09, I20–I25, I26–I27, and I30–I52) (9.74%), other diseases of the respiratory system (J95–J99) (7.35%) and secondary malignant neoplasm of other sites (C79) (6.25%). The top 10 comorbidities in male and female patients with BC as underlying cause of death are presented in **Figure 2** and **Supplementary Table S1**.

The top 3 comorbidities in 325 male patients with BC as all causes of death were lung cancer (C33–C34) (3.38%), secondary malignant neoplasm of independent (primary) multiple sites (C97) (1.85%) and heart diseases (I05–I09, I20–I25, I26–I27, I30–I52) (1.85%). The top 3 comorbidities of 261 female patients whose all cause of death including BC were secondary malignant neoplasm of independent (primary) multiple sites (C97) (1.92%), heart disease (I05–I09, I20–I25, I26–I27, I30–I52) (1.53%) and cerebrovascular disease (I60–I69) (1.15%). The top 10 comorbidities in male and female patients with BC as all cause of death are presented in **Supplementary Figure S1** and **Supplementary Table S3**.

### BC-Specific Premature Death

From 2005 to 2020, the YLL due to premature death from BC was 6,539.39 years, and the rate of YLL was 14.50/10^5^. YLL and the rate of YLL in men (3,604.24 years, 16.01/10^5^) were higher than those in women (2,935.15 years, 12.99/10^5^). In 519 BC-specific deaths, the main comorbidities were the diseases of the respiratory system (J00–J99) and the circulatory system (I00–I99), accounting for 14.84% and 4.82%, respectively. Moreover, 129 (24.86%) patients died due to BC-related metastases. The main metastatic sites of BC were the lung (10.21%) and liver (2.12%). The CMR, ASMRW, YLL, and YLL rates in different gender, periods, metastatic cancer, and comorbidity are detailed in **Table 1**.

### Age-Specific Burden of BC

A total of 386 (74.37%) elderly people aged over 60 years died from BC. The top 3 age groups with the highest CMR were ≥80 years, 70–79 years, and 60–69 years, which were 7.18/10^5^ person-years, 4.55/10^5^ person-years, 1.64/10^5^ person-years, respectively. Among them, the age group of 60–69 years had the highest YLL, with a loss of 1,440.79 years. The top 3 age groups with the highest rates of YLL were 70–79 years, ≥80 years, and 60–69 years, with rates of 42.77/10^5^, 37.68/10^5^, and 23.19/10^5^, respectively. The burden of BC in other age groups are shown in **Table 2**.

**Table 2:** Age-specific mortality and burden of bone cancer during 2005–2020.

Age (years group)	Deaths (N)	Proportion (%)	CMR (/10^5^)	YLL (years)	YLL rate (/10^5^)
0–4	0	0.00	0.00	0.00	0.00
5–14	11	2.12	0.37	321.16	10.89
15–29	24	4.62	0.33	662.84	9.20
30–44	23	4.43	0.23	569.66	5.60
45–59	75	14.45	0.64	1436.76	12.26
60–69	102	19.65	1.64	1440.79	23.19
70–79	149	28.71	4.55	1399.62	42.77
≥80	135	26.01	7.18	708.57	37.68
Total	519	100.00	1.15	6539.39	14.50

ASMRW, age-standardized mortality rate by Segi’s world standard population; CMR, crude mortality rate; YLL, years of life lost.

### Trends of Burden From BC

**Figure 3:**
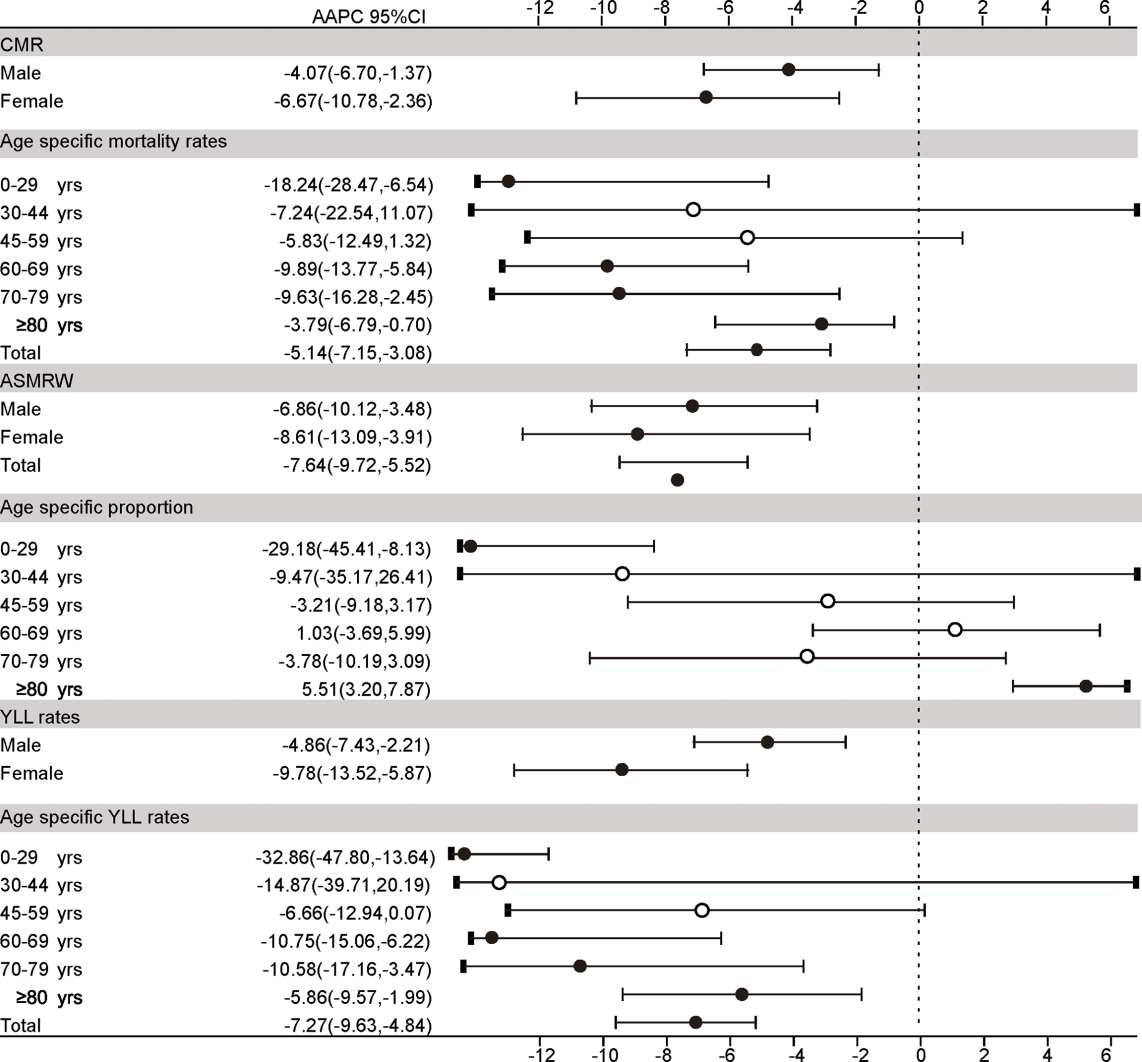
The trends in CMR, ASMRW, age-specific proportions, and YLL rate of persons with underlying cause of death from bone cancer in genders and age groups in Pudong New Area, Shanghai, China, 2005–2019. CMR, crude mortality rate (per 100,000); ASMRW, age-standardized mortality rate by Segi’s world standard population (per 100,000); YLL, year of lost, AAPC, average annual percent change; CI, confidence interval.

The long-term trends in CMR (AAPC = −5.14%), ASMRW (AAPC = −7.64%), and YLL rate (AAPC = −7.27%) were significantly decreasing in the total population from 2005 to 2020 (all *p* < 0.05). Details are shown in **Figure 3** and **Supplementary Tables S4**, **S5**.

The CMR, ASMRW, and YLL rate in female patients significantly decreased by −6.67%, −8.61%, and −9.78% per year, while those of male patients significantly decreased by −4.07%, −6.86%, and −4.86% per year (all *p* < 0.05). Details are shown in **Figure 3** and **Supplementary Tables S4**–**S6**. The age-specific mortality rates and YLL rates of aged 0–29 years and ≥50 years showed an obvious downward trend (all *p* < 0.05), except the age group of 30–44 years and 45–59 years. Details were shown in **Figure 3** and **Supplementary Tables S5**, **S6**.

The trends of age-specific proportion of death has shown that there was a significant decrease in the proportion of death aged 0–29 years (AAPC = −29.18%). Meanwhile, there was a significantly increase in the proportion of death aged ≥80 years (AAPC= 5.51%) (all *p* < 0.05). Details are shown in **Figure 3** and **Supplementary Table S7**. Interestingly, although the mortality of BC has decreased in the past decades, the proportion of BC-specific death within the total cancer-specific death in PNA dropped to a plateau without improvement since 2016 (**Supplementary Figure S2** and **Supplementary Table S8**).

### Quantitative Impacts of Demographic and Non-Demographic Factors on Mortality Rate

**Figure 4:**
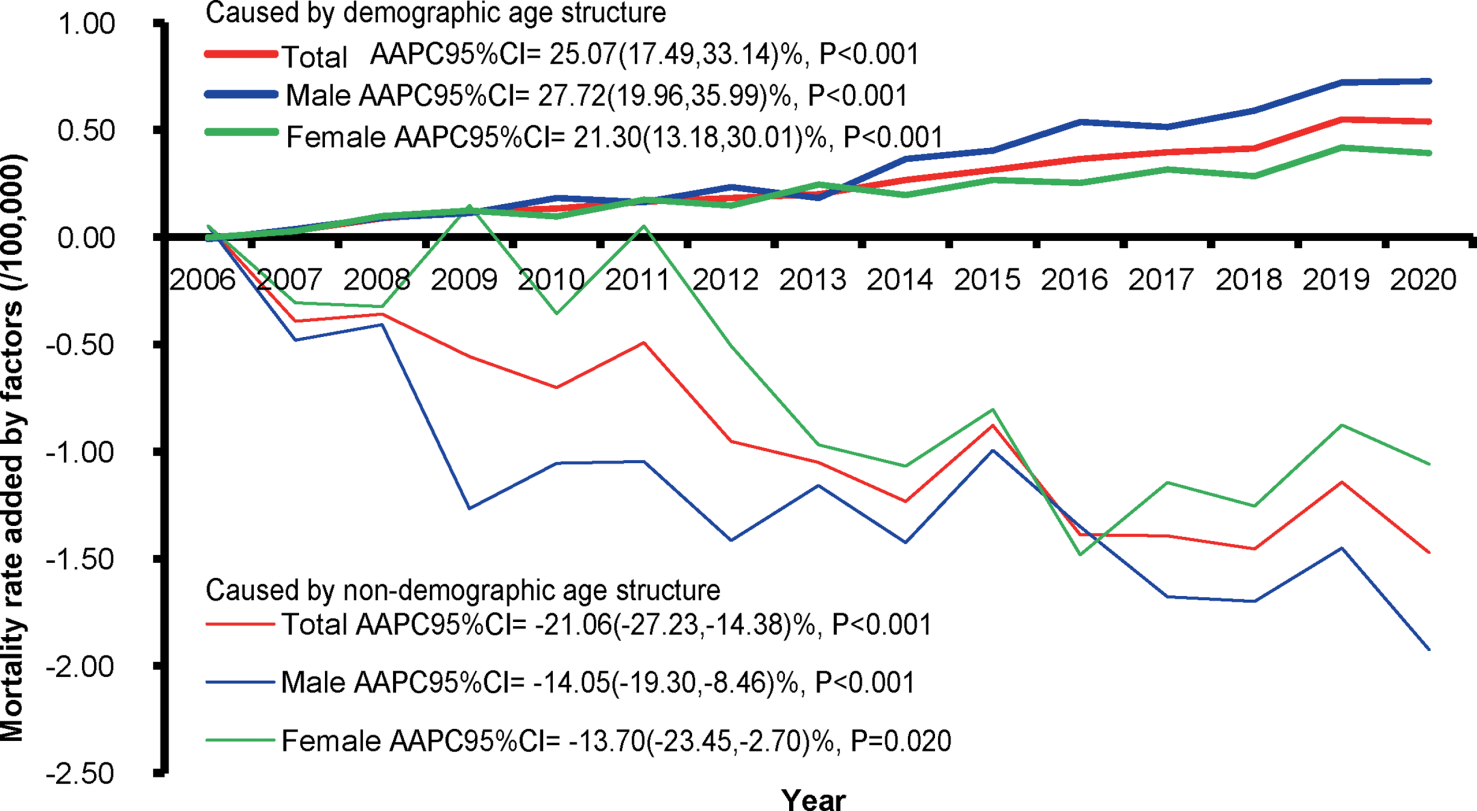
The increased rates caused by demographic and non-demographic factors and their proportion during the period from 2006 to 2020 compared with the crude mortality rate of bone cancer during 2005 in Pudong New Area, Shanghai, China. AAPC, average annual percent change; CI, confidence interval.

With the increase in the proportion of people aged ≥65 years in the local population per year (**Supplementary Figure S3**), increasing trends of CMR caused by demographic factors from 2006 to 2019 were observed, compared with the CMR in 2005 (**Figure 4** and **Supplementary Table S9**). A significant upward trend in the increase rate caused by demographic factors was noticed in the total population, with an annual percent change (APC) of 25.07% [(95% CI: 17.49%–33.14%), *p* < 0.001], whereas a significant downward trend was observed in the rate affected by non-demographic factors, with an APC of −21.06% [(95% CI: −27.23% to −14.38%, *p* < 0.001]. In male patients, the mortality rate affected by non-demographic factors decreased by −14.05% [(95% CI: −19.30% to −8.46%), *p* < 0.001], and the rate due to demographic factors increased by 27.72% [(95% CI: 19.96%–35.99), *p* < 0.001]. In female patients, the increased mortality rate due to non-demographic factors showed a downward trend [APC (95% CI) = −13.70% (−23.45% to −2.70%), *p* = 0.020], contrary to the rate due to demographic factors [APC (95% CI) = 21.30% (13.18%–30.01%), *p* < 0.001]. The concern is that, over time, the impact of demographic factors on the CMR in male patients was more obvious than that in female patients (*p* < 0.05).

## Discussion

As one of the fastest modernization districts of Shanghai, PNA has a gross domestic product (GDP) increasing from 10.1 billion RMB in 1992 to 789.8 billion RMB in 2015, with an average annual growth rate of 15.6%. The permanent residence of PNA has increased from 2.40 million in 2000 (14.92% of Shanghai) to 5.55 million in 2018 (22.9% of Shanghai) (4). Consistent with the reported data from the United States (3) and Europe (11), we found an obvious decrease in the mortality trend of BC during the urbanization process in PNA. Since the protocols and regimens for BC had minimal improvement in the past three decades (12), such reduction in mortality is likely due to the advance in the healthcare system, the medical infrastructure, and better adherence to the protocols. Furthermore, public education and awareness might also contribute to the prevention and early screening of BC. For instance, Holly et al. (13) observed that regular intake of mixed vitamin supplements during childhood decreases the risk of bone tumors (RR = 0.4; 95%CI: 0.1–1.4), and exposure to herbicides, pesticides, or fertilizers might increase the risk (RR = 6.1, 95% CI: 1.7–21.9). The reduction in other environmental hazards such as smoking during pregnancy (14) and ionizing radiation (15) in the lifestyle may also contribute to the observed trend of BC mortality. However, it should be noted that the proportion of BC-specific death within the total cancer mortality reached a plateau since 2016 (**Supplementary Figure S2**), indicating that the progress of BC has lagged behind that of the common cancer types in the past years.

It is noteworthy that there is a gender and age disparity in the observed decrease in mortality in the past 16 years. The female and younger age groups were the most significant contributors to the trend of mortality reduction, while the middle-aged and older populations benefitted less in the last decades. The potential causes might be twofold. On the one hand, it is reported that bone sarcoma patients aged 0–29 years (16, 17) and being female (18, 19) may have a greater response to current treatment protocols. By contrast, the optimal regimen for the elderly, especially those older than 65 years old, remains yet to be established (20). On the other hand, the age disparity in the improvement of mortality is also likely to reflect the refinement of current treatment strategies, which considerably reduce the short-term mortality of BC in the younger age group yet increase the chance of late recurrence, including the development of secondary malignancy, of BC. In line with this hypothesis, we found a relatively high percentage of secondary malignant neoplasm (12%) among the total mortality of BC in our cohort. These results reinforce the importance of long-term surveillance to reduce late mortality for those BC patients with short-term survivorship.

BC is well-known for its impact on the young- and middle-aged population. However, our results suggest that the proportion of BC patients younger than 30 years old was decreased, while that of the patients older than 80 years old increased in the total disease mortality during the past decades. The underlying reason for this trend might relate to Shanghai as one of the most and earliest aging societies in China. In 2008, PNA became an aging society, with 14.2% of residents aged over 65 years. In 2018, PNA was defined as a super-aging society, with more than 20% of residents aged over 65 years, and such proportion is still currently increasing (**Supplementary Figure S3**). Therefore, the elderly forms a distinct, understudied, and underserved group of BC burden in cancer care following the urbanization and socioeconomic development in Shanghai as an aging society (21). Importantly, although the overall mortality of BC demonstrated a downward trend, there was still a significant upward trend toward an increased mortality rate caused by the demographic factor (age), with an APC of 25.07% [(95% CI: 17.49%–33.14%), *p* < 0.001]. Given that older age (≥65 years) was known as a predictor of poor cancer survival in patients with overall bone sarcoma, osteosarcoma, chondrosarcoma (17), it is, therefore, an unmet medical need to take into account the age differences when designing future preventive and intervention strategies in Shanghai and other aging cities around the world.

There are several limitations to our study. First, due to the low number of BC as a rare cancer, the sample size of the present study is relatively low compared to common cancer types. Furthermore, one needs to interpret with caution when generalizing our findings to the total population of Shanghai, which is highly heterogeneous in terms of urbanization level, socio-economic status, healthcare infrastructure, etc. However, given that PNA area represents the most rapidly developing and transitioning part of Shanghai over the last decades, we proposed that our conclusions might be generalizable to those areas currently going or will go through the modernizing process in the suburban/rural part of Shanghai.

In conclusion, our study provides novel insights on the epidemiological characteristics and longitudinal dynamics of BC in a fast urbanization and transitioning society. We observed a significant reduction in BC mortality in past decades, yet with a clear gender and age disparity. As a disease affecting all ages, the burden of BC among the elderly might emerged as an understudied and unmet medical need in Shanghai, and other fast-transitioning cities worldwide.

## Data Availability Statement

The data presented in the study are available from the corresponding authors upon reasonable request and with permission of Center for Disease Control and Prevention of the Pudong New Area, Shanghai, China.

## Author Contributions

G-FM, Q-YB, and X-PL drafted and revised the manuscript. X-PL, G-FM, H-YZ, and Y-CC participated in the collection, analysis, and interpretation of data. Y-CC, YZ, H-YZ, and Q-YB contributed to data collection and suggestion for analysis. G-FM, X-PL, and J-HZ conceived the study and participated in its design and coordination and critically revised the manuscript. All authors read and approved the final version of the manuscript.

## Funding

This study was funded by a grant from special program for clinical research in the health industry of Shanghai Health Commission (20204Y0166 to J-HZ), a grant from the Key Specialty of Shanghai Pudong New Area Health Committee (PWZzk2017-08 to J-HZ), a grant from Shanghai Public Health System Construction Three-Year Action Plan Outstanding Youth Talent Training Program (GWV-10.2-YQ43 to Y-CC) and the Reserve Academic Leaders Training Program of the Pudong New Area Centre for Disease Control and Prevention (PDCDC-HBXD2020-05 to X-PL), and a grant from the Shanghai Municipal Health Commission (202140124 to Q-YB).

## Conflict of Interest

The authors declare that the research was conducted in the absence of any commercial or financial relationships that could be construed as a potential conflict of interest.

## Publisher’s Note

All claims expressed in this article are solely those of the authors and do not necessarily represent those of their affiliated organizations, or those of the publisher, the editors and the reviewers. Any product that may be evaluated in this article, or claim that may be made by its manufacturer, is not guaranteed or endorsed by the publisher.

## Supplementary Material

Supplementary Figure 1 The top ten of underlying causes of death of people died related to bone cancer in genders in Pudong New Area, Shanghai, China, 2005-2020.

Supplementary Figure 2 The proportion of people aged ≥65 years in genders in Pudong New Area, Shanghai, China, 2005-2020.

Supplementary Table 1 The sequence of underlying causes of death in 586 bone cancer related deaths.

Supplementary Table 2 The underlying causes of death in 67 deaths with bone cancer death from other diseases.

Supplementary Table 3 The sequence of all causes of death in 519 bone cancer specific deaths.

Supplementary Table 4 The distribution of definite pathological types in 519 bone cancer specific deaths.

Supplementary Table 5 The case number of death, CMR and ASMRW in gender during the period from 2005 to 2020 in Pudong New Area, Shanghai, China. ASMRW, age-standardized mortality rate by Segi’s world standard population; CMR, crude mortality rate.

Supplementary Table 6 The CMR and modeled CMR by Joinpont regression in age groups during the period from 2005 to 2020 in Pudong New Area, Shanghai, Chin. CMR, crude mortality rate.

Supplementary Table 7 The YLLr and modeled YLLr by Joinpont regression in gender and age groups during the period from 2005 to 2020 in Pudong New Area, Shanghai, China. YLL, the rate of years of life lost.

Supplementary Table 8 The proportion and modeled proportion by Joinpont regression in age groups during the period from 2005 to 2020 in Pudong New Area, Shanghai, China.

Supplementary Table 9 The increase value of CMR caused by demographic and non-demographic factors during the period from 2005 to 2020 in Pudong New Area, Shanghai, China. CMR, crude mortality rate.
