# Supplementary figures and images for: Patterns and Trends of the Mortality From Bone Cancer in Pudong, Shanghai: A Population-Based Study
Source: Front Oncol. 2022 May 16;12:873918. doi: 10.3389/fonc.2022.873918 (PMC9165546; doi:10.3389/fonc.2022.873918)

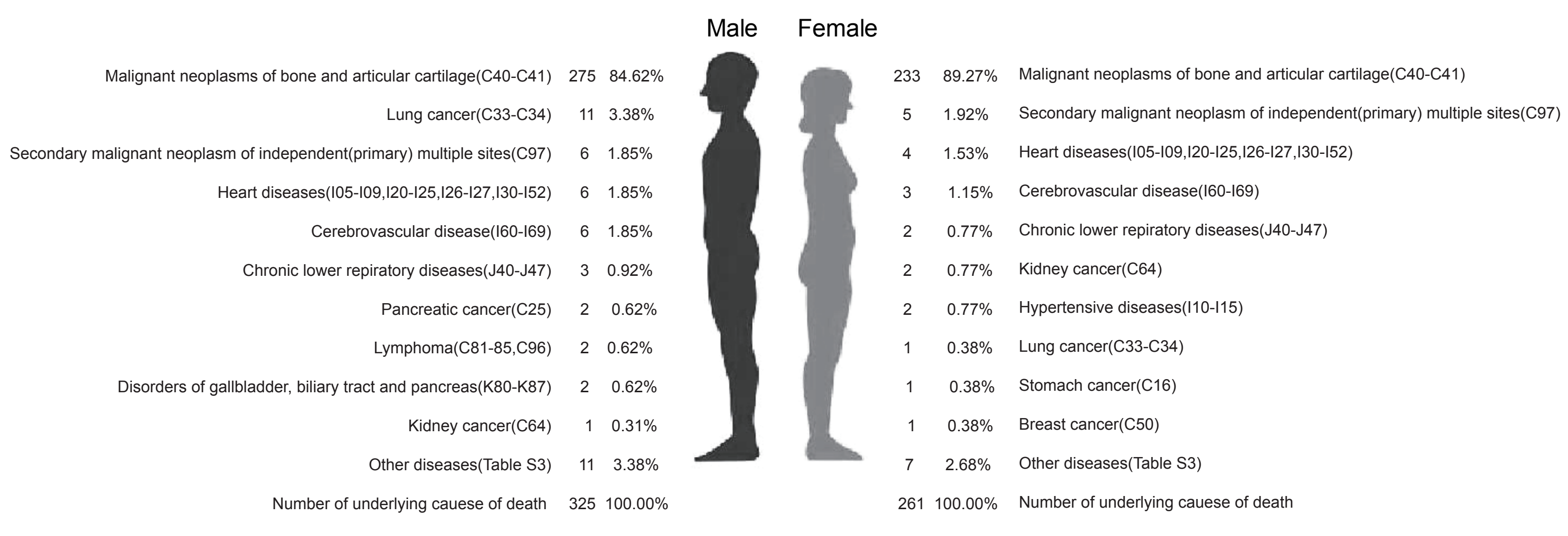

Supplement: Supplementary Figure 1 — The top ten of underlying causes of death of people died related to bone cancer in genders in Pudong New Area, Shanghai, China, 2005-2020. [file DataSheet_1.zip › Supplementary documents/Fig_S1.pdf]

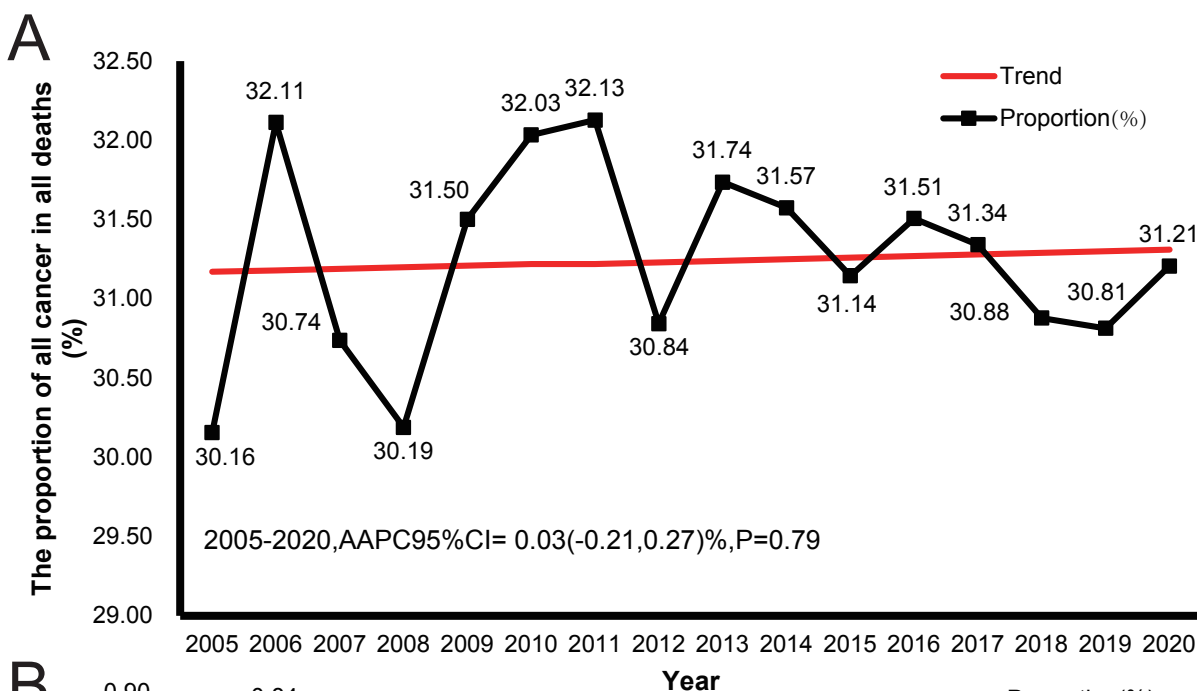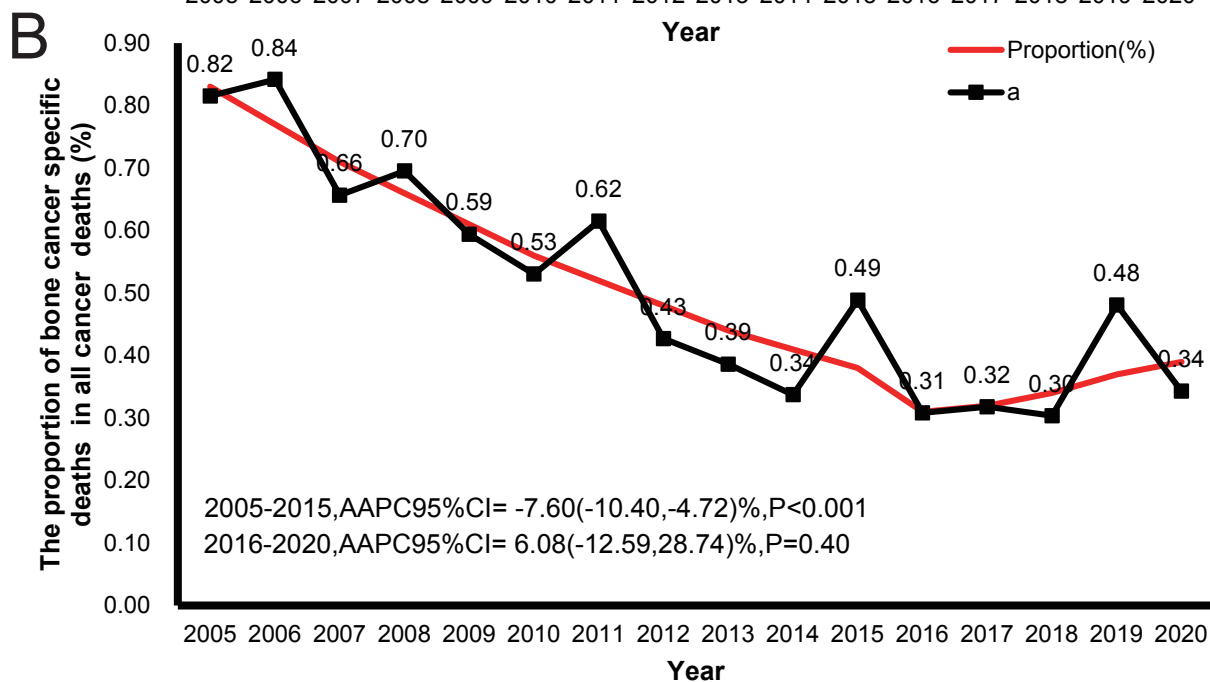

Supplement: Supplementary Figure 1 — The top ten of underlying causes of death of people died related to bone cancer in genders in Pudong New Area, Shanghai, China, 2005-2020. [file DataSheet_1.zip › Supplementary documents/Fig_S2.pdf]

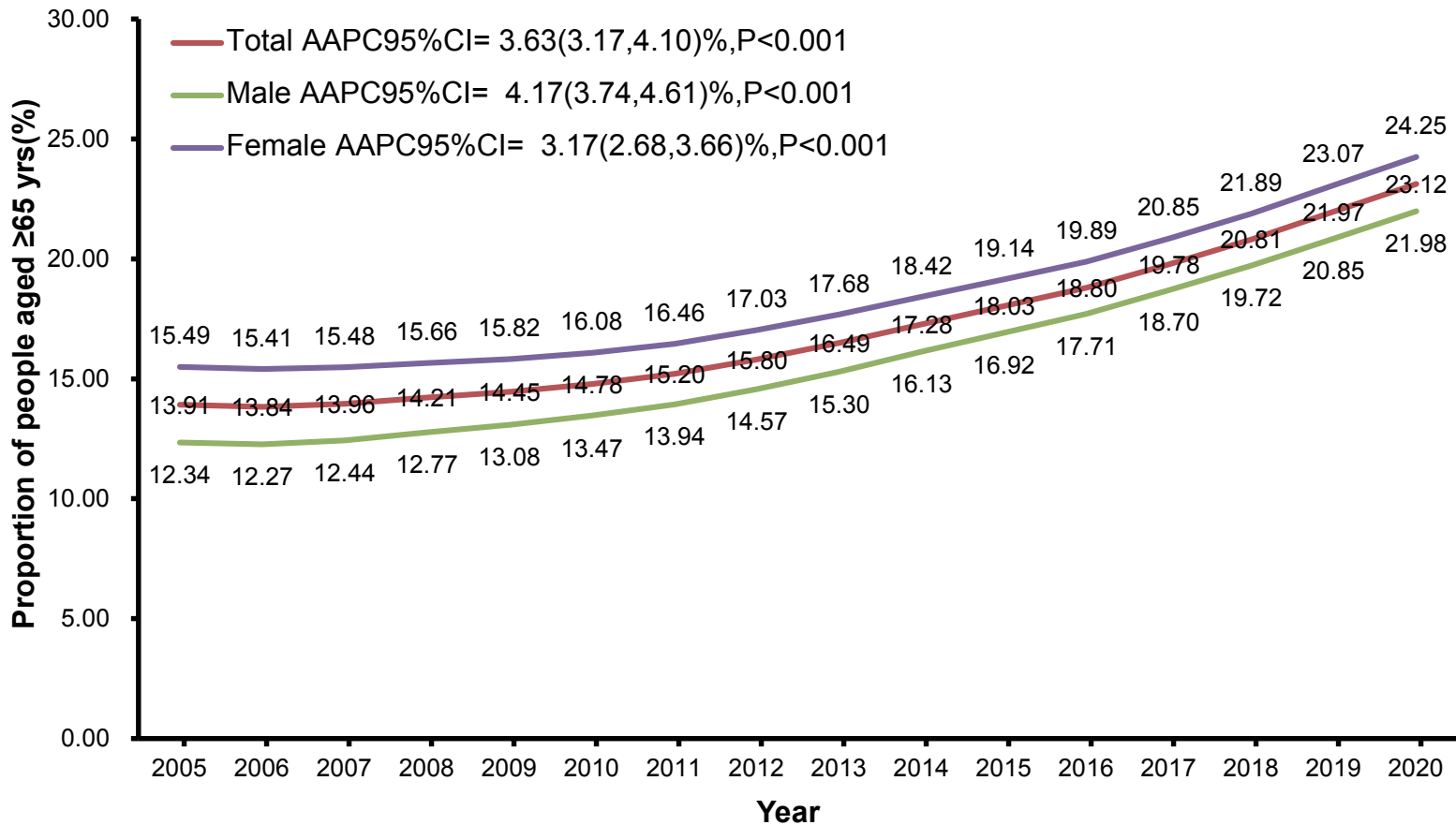

Supplement: Supplementary Figure 1 — The top ten of underlying causes of death of people died related to bone cancer in genders in Pudong New Area, Shanghai, China, 2005-2020. [file DataSheet_1.zip › Supplementary documents/Fig_S3.pdf]
